# Edible Coatings for Ready-to-Eat Products: Critical Review of Recent Studies, Sustainable Packaging Perspectives, Challenges and Emerging Trends

**DOI:** 10.3390/polym17030376

**Published:** 2025-01-30

**Authors:** Ina Bremenkamp, Maria José Sousa Gallagher

**Affiliations:** Process & Chemical Engineering, School of Engineering & Architecture, College of Science, Engineering and Food Science, University College Cork, T12 K8AF Cork, Ireland; 117106445@umail.ucc.ie

**Keywords:** edible coating, ready-to-eat seafood, eco-friendly innovation, novel food packaging system, shelf-life extension

## Abstract

If edible coatings are proven to control deterioration reactions by preventing chemical reactions, why aren’t they more widely used in industry applications, especially in the ready-to-eat food sector? This sector is a growing and emerging market and is interesting to diverse consumer groups. The potential of edible coatings as an innovative approach for more eco-friendly packaging systems should be further investigated. This article reviews the state-of-the-art developments of edible coatings for chilled RTE (ready-to-eat) food products as an area of growing interest and innovation, with a focus on sustainability, functionality, and costs. It discusses challenges associated with the use of edible coatings as eco-friendly packaging system in RTE food sector, including compatibility with food products, processing, shelf-life, storage conditions, cost, and regulatory requirements, and emerging trends, including biodegradable and eco-friendly coatings, shelf-life extension, active and intelligent coatings, and customization and personalization opportunities. Overall, while edible coatings offer many potential benefits in the RTE food sector, there are several challenges that must be addressed to ensure their successful implementation. Research and development efforts are needed to optimize the performance and stability of coatings while ensuring compliance with regulatory requirements and addressing cost concerns. The potential of edible coatings as eco-friendly packaging system should be further studied to highlight the full potential of edible coatings.

## 1. Introduction

More sustainable and novel food-packaging systems are in higher demand than ever before. In order to meet the world’s growing need for sustainable and high-quality food supply chains, two major challenges need to be addressed: food loss and waste reduction and packaging and packaging waste reduction, which are target issues of the sustainable development goals defined by the United Nations. Additionally, consumer demands such as mildly preserved, fresh, nutritious, safe, and convenient food products with enhanced shelf-life and longer retailing distances have driven the need for novel and innovative food-packaging [1]. 

Edible coatings have already been explored as packaging alternatives for food product groups such as vegetables, fruits, and fresh meat products [2]. However, can their potential be harnessed for RTE food products, which is a fast growing and emerging market, interesting for diverse consumer groups? Seafood products are particularly susceptible to spoilage and deterioration, and the use of edible coatings can help to extend RTE seafood’s shelf-life and improve their overall quality. Several studies have investigated the use of different edible coatings, with a focus on improving the safety, shelf-life, and sensory properties of products. Challenges associated with the use of edible coatings in the RTE seafood sector include the development of coatings that are compatible with different types of seafood products, as well as ensuring the safety and stability of the coatings over time. Additionally, regulatory requirements related to food safety and packaging materials must be addressed.

This critical review focuses on RTE seafood products and reports on current advances, challenges, and knowledge gaps that need to be addressed to enhance further developments in this area. Additionally, the potential of edible coatings for developing environmentally friendly responsible packaging systems for the RTE food sector is explored.

## 2. Edible Coatings

Edible coatings are thin protection layers that are directly applied to the product surface and can be consumed along with it [3]. They are made from natural film-formers that adhere to the product surface, creating a protective layer that can help to prolong the shelf-life by reducing or inhibiting natural chemical, biochemical or microbiological degradation processes caused by gas contact, gas diffusion or water transfer [4]. Essential properties of an edible coating include adhesiveness to the product, an invisible and uniform surface appearance, neutral or positive sensory attributes, a defect-free cover, and a composition that conforms to food regulations [5].

## 3. Recent Studies with Edible Coatings for RTE Products

Edible coatings have great potential for use in RTE food products. Yousuf et al. [6] have noted this potential, but limited research has been conducted on the use of such coatings in RTE muscle food products. The current state-of-the-art studies investigating the use of edible coatings in RTE seafood were reviewed. The investigation was broadened by including studies with edible coatings for muscle RTE products. The properties of single materials with and without additives as well as blends or multi-layer applications are explored. Some of the key findings are summarised in Table 1.

A chitosan coating with 1% (*w*/*v*) chitosan in 1% (*v*/*v*) acetic acid and 15% (*w*/*w* chitosan) glycerol, or a 1% (*w*/*v*) alginate coating with no glycerol and no crosslinking, showed the best performance in controlling the safety (microbial growth) and quality (water loss and lipid oxidation of a ready-to-eat (RTE) fish product under optimal and abused storage conditions [7]. The chitosan-coated samples showed the best performance, with a three-fold shelf-life extension compared to the uncoated products stored at 4 °C, suggesting that edible coatings have significant potential in enhancing the shelf life and safety of RTE fish products [7]. Moreover, the tested coatings demonstrated their ability to provide protective functions under abused storage conditions. Smoked sea bass fillets coated with chitosan or alginate packed in a vacuum bag and stored at 4 °C showed that a chitosan coating reduced the viable counts of mesophilic, psychrophilic, and anaerobic bacteria and additionally retarded oxidation [9]. The alginate-coated products improved the protection against oxidation but did not show an inhibition in bacteria growth. Cooked and peeled shrimps with a chitosan-thyme coating packed under MAP conditions in a tray with a film stored at 4 °C for up to 12 days showed a positive effect in inhibiting the growth of lactic acid and psychotropic bacteria [11] (. Retail maki sushi with salmon coated with chitosan, especially in combination with a low CO_2_—MAP packaging, was reported to increase the microbiological stability and preserve the colorimetric properties of the sample stored at abused temperature conditions (8 °C) and was recommend as a suitable hurdle technology for safety measures against temperature abuse in the food cold chain [8]. Spiced cooked RTE chicken meat with an edible crude nano-coating solution based on carboxymethyl chitosan, a water-soluble derivative of chitosan, and garlic aqueous extract inhibited the growth of microorganisms and delayed the rate of protein break-down and lipid oxidation [15]. Cold-smoked sunshine bass fillets coated with corn zein-based coating with lemongrass essential oil or nisin packed in bags with headspace air or vacuumed was studied with the focus on the inhibition of *Listeria monocytogenes*, showing that both coatings reduced the cell count in both studied packaging systems [10]. The nisin-treated samples were found to be the most effective additives against *L. monocytogenes* over the tested storage time of 42 days. Additionally, it was concluded that only the samples treated with corn zein-lemongrass essential oil in bags with headspace air were found to inhibit the growth of spoilage organisms. Other studies that focused on controlling *L. monocytogenes* in RTE meat products confirmed a positive effect of edible coatings with or without antimicrobials to reduce or even inhibit the growth [16,17,18] studied a chitosan coating on RTE meatballs; beside the inhibition effect on *L. monocytogenes*, other microbial populations were also counted, leading to a shelf-life extension of around 14 days. The conducted sensory test showed that the chitosan coating did not have a negative effect on the sensory characteristics of RTE meat balls. A packaging system consisting of an edible chitosan coating without additives and a synthetic polymer bag was tested for RTE roast beef under cooled storage [20]. The focus was to study the effect of the chitosan molecular weight and the solvent solution for the coating preparation on the reduction effect against *L. monocytogenes*. Chitosan coating prepared with an acetic acid solution were more effective in reducing *L. monocytogenes* compared to lactic acid solvent. The study also confirmed that chitosan coatings can be used to control *L. monocytogenes* on the surface of RTE roast beef products. The antilisterial activity of chitosan-based edible coatings in combination with vacuum packaging was also tested on cold-smoked salmon [13]. It was confirmed that a chitosan coating without antimicrobials was able to inhibit the population of *L. monocytogenes* on cold-smoked salmon during vacuum storage under refrigerated conditions, but tested chitosan coatings with antimicrobials had the greatest antilisterial activity. Jiang et al. [18] studied the effect of edible coatings for two RTE turkey products to control microbial growth with a focus on *L. monocytogenes*. Different polysaccharide coatings were tested as carrier materials as well as different antimicrobials in single and binary combinations. Alginate, kappa-carrageenan, pectin, xanthan gum, and starch displayed a good adherence and stability on the turkey products. The applied coatings had no negative effect on the texture, colour, smell, and overall appearance of the product. The different antimicrobial treatments had comparable efficiencies across the tested carrier materials, although pectin was slightly less effective than the others. Overall, it was shown that alginate-based antimicrobial coatings can improve the safety of poached and roasted turkey by inhibiting the growth of *L. monocytogenes* and reducing background microbiota levels throughout an 8-week storage period at 4 °C, though, to a different extent, depending on the selected antimicrobials. Another study focused on controlling the growth of *L. monocytogenes* on roasted turkey with a packaging system with edible antimicrobial coatings and vacuum packing into high barrier pouches [18]. Starch, chitosan, alginate, and pectin coatings with different antimicrobials were studied on sliced roasted turkey during cold storage and frozen storage. The results showed that pectin-based antimicrobial edible coatings hold promise in enhancing the safety of RTE poultry products and frozen storage has the potential to enhance their effectiveness. The antilisterial effectives of the tested polysaccharide-based carrier coatings with antimicrobials generally ranked in the order of pectin, followed by alginate, followed by chitosan and starch.

The investigated studies explored edible coatings for RTE fish products such as smoked fish bass, smoked salmon, cooked shrimps, and retail maki sushi. The studied RTE meat products were cooked chicken breast, fried meat balls, cooked turkey meat, and roast beef. Chitosan-based edible coatings with or without antimicrobial additives were primarily examined. Additionally, corn-zein, pectin, alginate, starch, gelatine, kappa-carrageenan, and xanthan gum were examined as carrier coatings for antimicrobials. The main focus of the investigated edible coating studies was the control of microbial growth with a special emphasis on *L. monocytogenes*, a common foodborne pathogen in RTE foods. It was shown that current packaging systems such as high barrier synthetic films, pouches, or trays were improved by the addition of a coating protection layer on the surface of the food product, extending the safety and quality of RTE products. Edible coating methods was intended as potential fresh-keeping methods for RTE products [15], but could also be a suitable hurdle technology for safety measures against temperature abuse in the food cold chain [8]. Only a few studies focused on other protection properties as fat oxidation or water loss, beside the microbial degradation [9,14]. The fact that an edible coating can offer multiple packaging functions by also protecting physical, biological, or chemical quality changes of food products is under-investigated in the case of RTE food products. Controlling multiple degradation processes can increase the potential use of a coating as an integrated part of a packaging system. It was noticed that the tested edible coatings were developed as an added protection layer to the existing primary package but not with the focus on overtaking functions of the current packaging system. The synergy between an edible coating and an outside layer can be of interest for an eco-packaging design option and needs further investigation. Additionally, it was noticed that only composite coatings with a polysaccharide carrier material and antimicrobials were studied. Other potential methods, e.g., multilayer coatings or blends, for improving the coating performance were not studied with RTE seafood products.

## 4. Challenges and Emerging Trends

**Figure 1:**
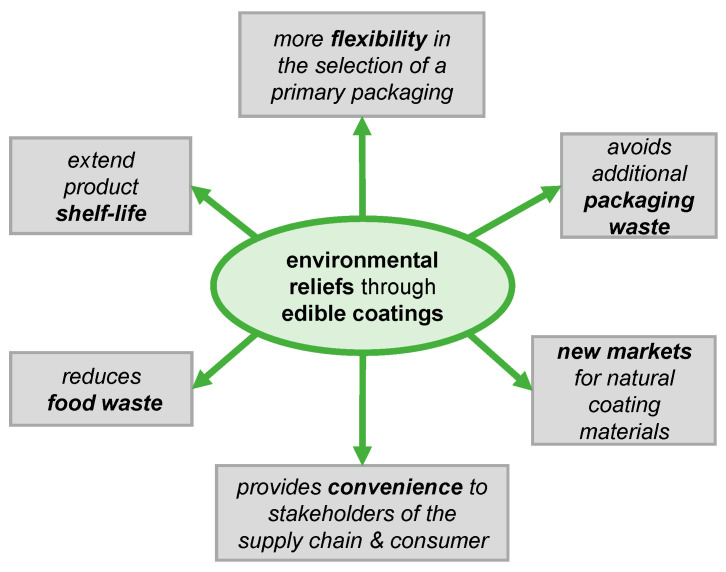
Overview of challenges and opportunities of biobased edible coatings.

To explore the full potential of edible coatings for RTE products, the concept was reviewed under a holistic approach. Potential challenging aspects, emerging trends and opportunities were discussed (Figure 1). Sustainability aspects of edible coatings and coating integration requirements for being part of the food product were investigated. Additionally, strategies to improve coating performance, application methods and methodologies for testing the coating performance were reviewed.

### 4.1. Can an Edible Coating Provide an Eco-Packaging Solution?

Food packaging is complex and multidimensional and plays a crucial role in ensuring the quality, safety, and security of food products. Food packaging offers several functions and services to products such as protection, containment, communication, and convenience [23]. One important aspect of food packaging is its protective function, which helps to prevent degradation processes, such as mechanical, physical, chemical, or biological damage. Depending on the food and its characteristics, degradation processes vary, and the emphasis of packaging requirements is placed on different prioritized protective functions [24,25,26]. However, in addition to providing protection, food packaging also has an important role to play in minimizing environmental impact. An environmentally responsible food-packaging system involves a synergy between reducing food losses and waste from production to consumption, as well as using packaging materials that require minimal resources and generate minimal waste [27]. RTE muscle products are highly perishable and require specialised packaging to ensure their safety and quality. However, traditional multilayer packaging systems, which combine different materials to achieve specific packaging properties, can be difficult to recycle and contribute to environmental pollution. The approach of multilayer packaging offers strengths and weaknesses [28,29,30]. Multilayer food packaging is a lightweight solution but is identified as an obstacle on the way to achieving circularity, as flexible packaging is hardly recycled [28,29,31]. The dependence on fossil raw materials is an additional environmental burden. A good balance between eco-friendliness and the preservation of shelf-life is crucial for food-packaging systems. While incorporating an edible coating into a packaging system can incur additional expenses, such as those related to coating development, application, and product–coating interactions, it is worthwhile to examine the potential benefits of using an edible coating as a primary packaging system. An edible coating is sourced from biobased materials, whereby the environmental impact of an edible coating depends on the selected coating material, as discussed in Section 4.2. An edible coating is designed to be consumed with the product and therefore no additional waste is generated. Often only a small amount of coating material is needed to cover the food product surface and supply a protection function. The type of polymer that can be used depends on the product properties, and the supply chain requirements, usually cold chain conditions for RTE muscle/seafood products.

Numerous studies have investigated the use of edible coatings in combination with traditional synthetic primary packaging, with a focus on increasing product shelf-life and reducing food waste by reducing degradation processes such as microbiological growth, but also increasing the product robustness against temperature changes during the supply chain [8]. Studies focusing on the coating performance without an external layer have shown that the coating alone can protect and extend product shelf-life [14,21]. This could indicate that an edible coating in combination with an external packaging provides protection even when the integrity of the external packaging is broken at the consumer stage. This approach can support the use of larger purchasing units, reducing packaging waste and providing convenience to the consumer by reducing shopping frequency, and offering more flexibility in consuming the product.

An under-investigated concept related to edible coatings is the use of edible coatings as an integrated part of the primary packaging. Hereby, the focus is on developing environmentally friendly packaging systems by reducing the environmental impact of the external layer, instead of increasing the shelf-life with a coating. The concept is based on the ability of a coating to overtake packaging functions of the external packaging by controlling single or multiple degradation processes, e.g., water loss, fat oxidation, or microbiological growth, with the aim of reducing the requirements of external packaging and potentially opening the possibility of selecting more environmentally friendly materials. Viable options could be mono-material applications, more easily recycled materials, or bio-based materials. Further investigation is needed into the concept of edible coatings as an integrated part of primary packaging systems, including applied packaging and theoretical modelling studies.

**Figure 2:**
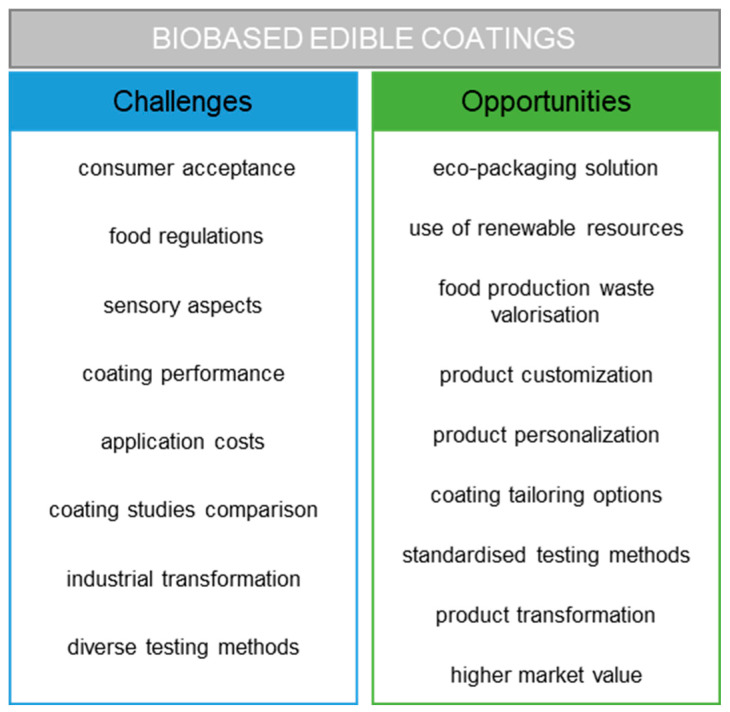
Challenges and opportunities of eco-food packaging systems with edible coatings.

Overall, a packaging system consisting of an edible coating and an external layer as primary packaging could lead to a more sustainable packaging system by reducing food waste, using bio-based (edible) packaging materials, providing more flexibility in the external packaging selection, providing convenience to the consumer, and increasing shelf-life, leading to more flexibility for producers, wholesalers, and retailers in the supply chain (Figure 2). An edible coating cannot stand alone as primary packaging as it does not protect from contamination during storage and transportation and does not provide containment or communication, but it should be seen as an added or integrated part of the packaging system. Edible coatings in combination with an external layer are described as a fast and cost-effective technology with a substantial industrial potential that is easy to implement [8]. An edible coating could meet consumer demand for high-quality food products in combination with the environmental need to reduce packaging waste [32]. Nevertheless, more studies are needed considering an edible coating as an integrated part of the packaging systems to reduce the environmental impact of the packaging system with a similar shelf-life, instead of an added part to extend the product shelf-life. The full potential of edible coatings as drivers for sustainability has still not yet been explored. Life cycle assessments studies should be used to provide evidence of the developments and functions of an edible coating as an eco-packaging system.

### 4.2. Developing Strategies for Edible Coatings

**Figure 3:**
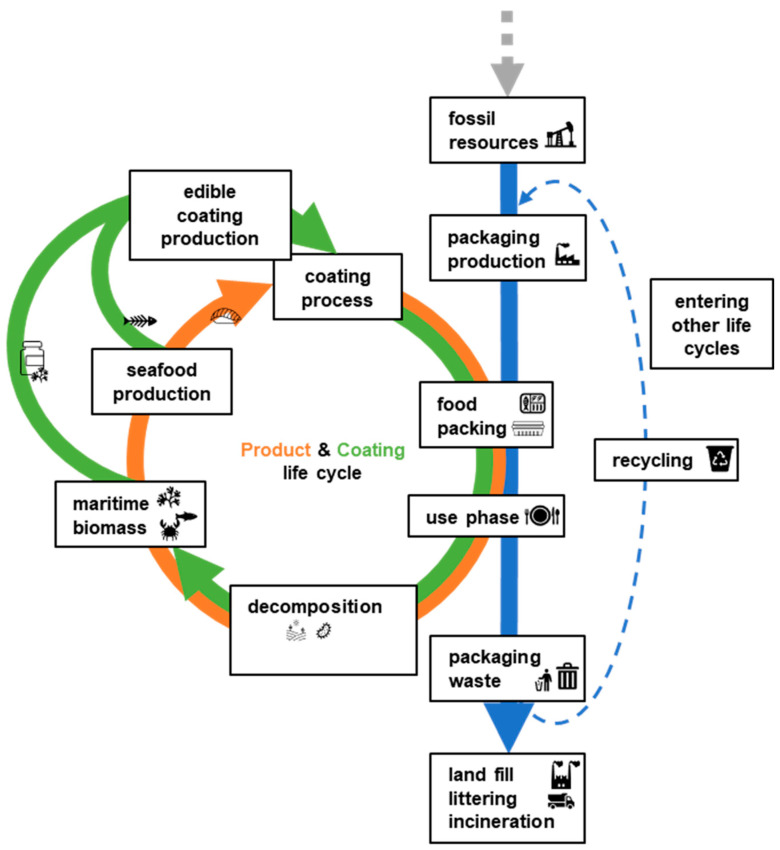
Overview of life cycle for a food product, edible coating, and external packaging.

RTE muscle foods are highly perishable products intended for direct consumption. These products are often transported and stored in chilled supply chains and have a limited shelf-life due to microbiological and biological reactions as well as chemical degradation. As a result, ensuring food safety is a critical factor. In addition to this, it is important to control common deterioration reactions in RTE muscle foods such as water loss and fat oxidation to prolong product quality and safety. When selecting coating materials, it is important to consider their ability to provide characteristic protection functions, as well as nutritional and sensory aspects as discussed in Section 4.3. Moreover, it is essential to investigate the potential for environmental conservation and conscious handling of available resources in the selection of coating materials. A circular economy principle supports a design that includes options to make use of resources, materials, and products after the use phase [33]. Avoiding a linear consumption pattern is essential, often used for many global resources where the focus lies on the consumption of resources without planning further use at the end of life. Prioritising circular aspects will lead to supporting sustainable aspects during the design phase [34]. A wide range of natural edible coating materials are available from land or water resources. In the case of RTE seafood products, exploring coating materials from the maritime environment was favoured to support an integrated circular life cycle, in contrast to the often linear life cycle of a synthetic packaging material (Figure 3). Therefore, it is of value to explore potential marine-based coating materials by considering available biomass, materials from waste streams, their resource availability, or competition with food supply.

An overview of generated products from maritime biomass, including finfish-based, shellfish-based, and plant-based biomass, can be found in Olsen et al. [35]. Interesting products for edible coating materials include, among others, polysaccharides sources from seaweeds such as alginate, carrageenan, and agar, sources from crustacean shell waste such as chitin and chitosan, or sources from algae such as cellulose and starch (Table 2). Interesting protein sources are collagen and gelatine, and lipid sources are fish oil from fin-fish by-products. Additionally, minerals and other functional ingredients can be of interest to improve the coating properties and improve the nutritional value. Valuable polymers such as chitin and chitosan are being extracted and modified from crab waste for potential coating applications and new markets avoiding environmental pollution. Waste products that are not valorised can end up in landfills, soil dumping, or be discarded in seawater, resulting in major surface pollution and unpleasant smells in coastal areas and contributing to major environmental concerns [36]. Various marine by-products have been researched and developed for many years for different applications. While some are successfully implemented and established on the market, such as chitosan production from crustacean shells, others have been limited due to several drawbacks, particularly for fin-fish by-products. These limitations may be due to overestimation of market possibilities, inadequate amounts of high-quality by-products available on a regular basis, excessive costs of isolating specific components often present in lesser amounts in the by-products, or cheaper and more stable production methods using genetically modified microbes or chemical synthesis. Furthermore, regulations and documentation costs for upcycling by-products for human consumption may also pose challenges [35]. Marine plants are a fast-growing sustainable recourse for biomass which can be produced without significant environmental impacts [37]. They are considered next-generation bio-based materials [38,39] and have advantages such as minimizing or avoiding competition with arable land and nutrients used for conventional agriculture, reducing competition for limited freshwater supply, utilizing wastewater, reclaimed water, and saline water, and recycling carbon dioxide during growth by carbon dioxide fixation and oxygen production [40]. Additionally, they have an extensive environmental tolerance [41,42].

The available biomass from aquatic resources includes high-value polysaccharides, proteins, lipids, and minerals that can be used for the development of edible coating materials. Some materials, such as chitosan, alginate, agar, carrageenan, fish oil, and fish gelatine, are already implemented into the current market, while others, such cellulose or starch from algae, are still at the research level and need to be scaled up to reach a competitive market value.

### 4.3. Edible Coatings: An Integrated Part of Food Products

An edible coating is an integrated part of the food product intended for direct human consumption and ideally unnoticeable to the consumer. Thus, in addition to protection functions, organoleptic and nutritional aspects and food regulations play a significant role for edible coatings [5]. Specialized edible coatings can be designed to have specific protection functionalities, such as antimicrobial properties, or oxygen scavenging, as in so-called active coatings. Additionally, coatings find use within intelligent packaging systems to monitor freshness and enable real-time monitoring of the quality and safety of food products during whole supply chain. Active and intelligent coatings are of high research interest.

The mouthfeel, texture, taste and aroma throughout the whole shelf-life of the product should be considered when developing an edible coating to increase consumer acceptance and market transformation. Additional values of edible coating are customization and personalization potential. Consumers are seeking more personalized and customized products, and edible coating can provide a platform for product differentiation. For example, coatings can be customized with different flavours or colours to enhance the sensory experience of the consumer. Another emerging trend is the use of edible coatings as carriers for nutraceuticals such as vitamins or minerals, allowing product tailoring to meet the specific demands of consumer groups such as elderly people. Difficulties may arise due to interactions between coatings and product components, which could impact organoleptic or nutritional aspects. These aspects are conditional for the use of an edible coating for industrial products.

The use of edible coatings in the RTE food sector is subject to regulatory requirements related to food safety, labelling, and packaging materials. Compliance with these requirements can be challenging and time-consuming [6]. Additionally, further research is required to explore consumer views about edible packaging. By better understanding acceptance and hurdles, relevant communication can be developed for products with edible coatings. A drawback of the development and implementation of edible coatings into the RTE food sector are additional costs due to coating development, implementation, material costs, or equipment investments. Nevertheless, additional direct costs can be justified by providing additional product and market value through product quality and safety or product differentiation from competitive products. In the realm of studies on edible coatings for RTE seafood products, standard methodologies considering both protection properties and sensory aspects should be further implemented to support the interdisciplinary fields of coating development and its implementation into industrial application.

### 4.4. Strategies to Improve Coating Performance: Active and Intelligent Coatings

The more effective an edible coating is at protecting the product, the less reliance there is on the external packaging. To optimise the protection function for a specific food product, it is important to consider different options for tailoring the edible coating [43]. Using a single material may only provide limited protection; therefore, composite or multilayer coatings can combine the unique properties of different materials and form a protective layer for different critical quality parameters simultaneously [6,43,44].

Coating properties can be tailored by blending different materials (composite coatings), either from the same material group, such as polysaccharides, proteins, or lipids, or different groups [43,45]. However, the latter case is more common, since the individual protection properties are more diverse [6]. For instance, hydrocolloids, known for offering a good gas barrier, can be blended with a lipid component to improve water barrier properties and enhance mechanical and structural coating properties [3,46]. Nevertheless, blending materials has its limitations, such as difficulties in forming a homogeneous coating solution, turbidity due to small light-breaking particles distributed in the solution, and the formation of aggregates or phase separation.

Crosslinking is an irreversible change of the polymer structure through a chemical reaction and is another interesting way to tailor coating formulations. It is often applied through a double coating process. For instance, sodium alginate can be cross-linked with calcium carbonate to form a water insoluble gel coating. But other materials are also cross-linked to tailor their coating properties, such as chitosan coatings [47].

Another strategy is the addition of synthetic or natural additives such as antioxidants, antimicrobials, gas scavengers, nutraceuticals, plasticizers, or nanoparticles. Active coatings are defined as coatings that interact with the food to deliberately incorporate components that would release or absorb substances into or from the packaged food or the environment surrounding the food to extend the shelf-life of packaged food [48]. The research focus is mainly on using natural compounds to eliminate potential health concerns and increasing consumer acceptance. Antimicrobial agents such as nisin, lysozyme, and essential oils, antioxidant agents such as gelatine hydrolysates [43,49,50], and nanoparticles such as nanocellulose or clay [51] have been studied. However, the use of additives has limitations, such as application costs, their intensive aroma or potential toxicity [51], and the potential migration of nanoparticles to the human body [52].

A different approach to improving coating performance is the use of multi-layer coatings. Composite coatings involve blending materials to form a stable mixture, while bilayer coatings can be prepared through layer-by-layer or unstable mixture techniques [53]. Multi-layer coating systems form layer-by-layer structures on the product surface and have been studied for the application of lipid-based coatings [43,53]. Multi-layer coatings of chitosan and alginate coatings have been used on fresh cut watermelon to combine coatings with opposite-charged polymers [54]. Lipid-based bilayer coatings based on different lipid components or lipid and hydrocolloid layers have been reported to provide a much higher moisture barrier compared to composite coatings of the same materials [55]. While bilayer coatings can add up the properties of single material coatings [56], they also have drawbacks, such as forming a thicker coating layer that may lower consumer acceptance and require an additional processing step, which can be a hurdle for implementation in industrial applications.

Therefore, developing coatings that can simultaneously control multiple degradation processes would increase their protection effect and potentially their wider application. In addition, life cycle studies are highly recommended to further highlight the potential of edible coatings to improve the environmental impact of food-packaging systems [57]. A circular economy approach considered during the development of an edible coating can help to create new markets for waste streams and biomass of the maritime industry.

### 4.5. Coating Application Methods

Biobased coatings can be applied to the product surface by dipping, spraying or vacuum impregnation. For more detailed information, please refer to Senturk Parreidt et al. [58] and Andriani & Handayani [43]. In all methods, the first coating formulation must be prepared by dissolving the biopolymer in a solvent, followed by addition of components, homogenization, and a degassing step (filtration, ultra-bath, centrifugation or resting). Depending on the coating materials, other steps like heating may be necessary to ensure structural breakdown or improve dissolving properties.

At the laboratory scale, dipping is the most used coating method [58]. The product is coated by dipping into a coating formulation for a defined time, then drained to remove excess solution, and finally dried by evaporating the solvent.

The coating process is typically repeated with the same or a different solution to ensure a continuous film formation on the surface of the product. Dipping creates an even coating of products with complex and rough surfaces. The coating thickness can be controlled to some extent with the viscosity of the coating formulation [59]. However, compared to the spraying methods, coatings applied with dipping are generally thicker [58]. Difficulties may arise when coating hydrophilic product surfaces with a hydrophobic coating solution. A layer-by-layer coating method can solve this issue by promoting physical and chemical bonding between oppositely charged materials [43,58]. One drawback of the dipping method is the potential cross-contamination when multiple products are dipped into the same coating solution, which can result in product components dissolving into the coating solution.

Spraying is a coating method where the coating solution is distributed as droplets over the targeted food surface area through a nozzle. This method has several advantages, such as the need for less coating solution due to applied high spraying pressure, creating a uniform coating with a controlled thickness, enabling temperature control of the solution, and being suitable for multilayer applications. Additionally, this method avoids contamination of the coating solution, making it possible to work with a larger product surface area. The spraying system’s coating efficiency is affected by various factors, such as the properties of the coating solution (density, viscosity, and surface tension), operational conditions (flow rate, air pressure, etc.), and system conditions (nozzle design, spray angle, etc.). It is important to consider the spray-flowing characteristics of the coating solution, where a low viscosity of the coating solution is preferred. However, many biobased polymers have a high viscosity at low polymer concentration, which can be an issue. The combination of dipping and spraying methods is possible, and it provides advantages such as a thinner layer, better coverage, and controlled thickness [60].

Vacuum impregnation is a third coating method that involves the same dipping and draining steps as the dipping method. However, in vacuum impregnation, the samples are coated in a vacuum chamber to ensure rapid and direct migration of the coating material into the porous tissues of the samples. The method works by removing gas and liquids from the pores of the product and replacing them with coating solutions under restored atmospheric pressure [61].

After the coating application, the samples were often allowed to dry before being further packed or stored to ensure complete coating coverage around the product. Drying at room temperature, during cold storage, or at higher temperatures under laminar or turbulent air circulation was reported.

Only a few research papers on edible coatings for RTE seafood or related products using spraying or vacuum impregnation method were found, with most studies applying the dipping method. Further investigation of the spraying methods is recommended to bridge the gap between research and industrial applications.

### 4.6. Coating Performance Measurement Methods

Important coating properties include adhesiveness, water barrier, gas barrier, antimicrobial properties, antioxidation properties, appearance, taste, and texture. The purpose of this section is to provide an overview of methodologies used to validate and compare coating performances (Table 3).

Viscoelastic properties of the coating formulations, such as pH value, elastic modulus, viscosity modulus, solubility, phase angle as a function of temperature, and electrical charge, are important in ensuring easy handling, application, and increasing industrial feasibility [3,14].

When the coating is applied to the product, the adhesiveness, coating thickness, and coating integrity properties are of interest. The adhesiveness of the coating material on the product depends on the nature of the material and on the number of interactions or bonds between the coating and support [62]. Some researchers investigated coating adhesiveness by measuring the contact angle of the product of interest before and after coating [4]. In addition, the coating integrity on the surface and the penetration of the coating can be analysed through imaging analysis. The coating thickness can be measured with the aid of a scanning electron microscope (SEM).

Coating barrier properties against water and gas can provide relevant information about the coating performance. To evaluate the barrier efficacy of coating materials, either the barrier properties of dry-casted films (permeability) or the barrier measurements of the coating directly applied onto the product (resistance) are determined. However, measuring the barrier properties of casted coating films has the disadvantage that the results are often difficult to transfer to the barrier properties of the coating material applied to the product, as they can be highly influenced by food contact [13,17,21,63]. To evaluate the relative water loss of coated products, the rate of water loss (g/h) is measured or the water vapour resistance of the coating (s/cm) is determined to compare the performance of different coatings, considering parameters as water activity, relative humidity, water loss rate, surface area, saturation pressure, and storage temperature [64,65]. Although research on the effect of barrier properties on edible coatings with the product is still limited, this method has been applied to diverse coatings and food products [56,66,67,68]. This method allows for comparing different coating materials and assessing their efficiency on various products. Another direct method for studying the gas barrier of a coating is by measuring the gas exchange rates (e.g., O_2_ or CO_2_) of the coated product with the headspace volume in a closed system [4].

The effect of the coating on the degradation process of food samples can be assessed through microbial, chemical, and physical analyses. Antimicrobial properties can be evaluated by measuring the growth of bacteria, yeast, and mould, with the specific strains selected based on the food product and storage conditions of interest. Physical-chemical deterioration can be monitored by measuring factors such as water loss, water activity, nitrogen degradation, and fat oxidation (peroxide value, malondialdehyde value, total volatile basic nitrogen, or trimethylamine-nitrogen), as well as pH changes and adenosine triphosphate breakdown during storage [69]. Changes in the appearance of the product can be measured using a colorimeter, while the texture can be assessed with a texture analyser that measures the hardness, elasticity, cohesiveness, adhesiveness, chewiness, and gumminess of the coated and non-coated products [14]. Sensory evaluations can also be conducted to study the appearance, odour, texture, firmness, colour, taste, juiciness, and flavour of coated products.

Various methodologies have been used in the literature to measure the coating performance [43]. However, standard methods for measuring the coating integrity, penetration, and barrier properties are still lacking. Additionally, the comparison of coatings applied to different food products is limited as the coating properties are highly affected by the product or interactive properties. Direct methods for studying the coating properties are limited, and indirect measurements are still prevalent in coating studies.

## 5. Conclusions and Future Trends

The consumer demand for healthy, safe, and convenient food products, as well as sustainable food-packaging solutions, is driving the development and market implementation of RTE products packaged with eco-innovative packaging solutions. This review investigated the state of the art of using edible coatings for RTE seafood products.

An important aspect driving the use of edible coatings is their sustainable potential, such as the fact that they generate no waste generation, are bio-based, or improve food shelf-life. Life cycle studies are highly recommended to further highlight the potential of edible coatings to improve the environmental impact of food-packaging systems. A circular economy approach considered during the development of an edible coating can help to create new markets for waste streams and biomass of the maritime industry.

Reviewed studies show promising results for the use of edible coatings for improving product safety and shelf-life. Edible coatings have been highlighted as potential methods for fresh-keeping RTE seafood products and improving the sensitivity of products against temperature abuses in the food cold chain. Developing coatings that can control multiple degradation processes simultaneously would increase their protection effect and their potential wider application.

Another hurdle identified for the use of edible coatings for industrial products is the consideration of food appearance, regulations and labelling issues, and consumer acceptance. Some market value opportunities related to edible coatings are product customization and personalization options. Challenges are related to counter-effects between protection properties and sensory properties. Developing standardised methodologies for testing edible coatings could improve the current difficulty of comparing edible coating performances among different studies.

Overall, the RTE food sector is an area of growing interest and innovation for edible coatings, with a focus on sustainability, functionality, and customization. The development of edible coatings for RTE seafood/muscle products is an interdisciplinary challenge that needs to take into consideration technological, engineering, environmental, and consumer aspects. While edible coatings offer many potential benefits in the RTE food sector, there are several challenges that must be addressed to demonstrate their compatibility with food products and optimize their performance and stability while ensuring compliance with regulatory requirements and addressing cost concerns.

## Figures and Tables

**Table 1:** Edible coatings for Ready-To-Eat fish and meat products.

Product	Edible Coating Material and Coating Method	Additional Packaging	Storage Conditions	Source
Ready-to-eat seafood	Chitosan in acetic acid and glycerol, or alginate with no glycerol using dipping method	-	4 and 14 °C	[7]
Retail maki sushi	Chitosan coating applied using spraying method	Packed under MAP conditions	4 and 8 °C	[8]
Smoked sea bass fillets	Chitosan, alginate with glycerol, applied using dipping method	Packed under vacuum	4 °C	[9]
Cold-smoked bass fillet	Corn zein-based coating with lemongrass essential oil or nisin applied using spraying method	PVC bags with headspace or in vacuum	4 °C	[10]
Cooked and peeled shrimps	Chitosan, glycerol, Tween 80 coating incorporated with 0.5% of oregano and thyme EO applied with dipping method	Packed under MAP conditions (40% CO_2_/60% N_2_) in polystyrene trays with a PET/PVdC/PE film	4 °C	[11]
RTE squid rings	Oregano and thyme essential oils with dipping method	Packed under MAP conditions (70% N_2_, 25% CO_2_, 5% O_2_) in film bag	4 °C	[12]
Cold smoked salmon	Chitosan coating with and without antimicrobials with spreading method	Vacuum packed	4 °C	[13]
Fish patties (RtC)	Chitosan—fish gelatine coating applied using dipping method	-	2 °C	[14]
Cooked chicken meat	Carboxymethyl chitosan with garlic aqueous extract applied using dipping method	Open packaging pouch	4 °C	[15]
Fried bovine meatballs	Chitosan (HMW) with dipping method	EPS trays in plastic food bag	5 °C	[16]
Deli turkey meat	Chitosan with antimicrobials (lauric arginate ester or nisin or in combination) with spreading method	Vacuum pouches	10 °C	[17]
Roasted sliced turkey	Pectin, alginate, chitosan, starch with different antimicrobials with spreading method	High barrier pouches, vacuum	4 °C	[18]
Deli turkey and in pouched cooked sliced turkey	Polysaccharide carry materials (alginate, kappa-carrageenan, pectin, xanthan gum, starch) with different antimicrobials with spreading method	Vacuum packed	22 °C, 4 °C	[19]
Roast beef	Chitosan coating (different molecular weights and solvents) with dipping method	Bagged	4 °C	[20]
Precooked beef patties	Wheat gluten, soy protein, carrageenan, and chitosan coatings with dipping method	-	4 °C	[21]
Dry sausages	Alginate, polyglycerol esters of fatty acids, pea protein, and collagen with extrusion method	-	13 °C	[22]

**Table 2:** Overview of maritime materials for edible coatings.

Product Category	Product Name	Maritime Source	Market Implementation
Polysaccharide	Alginate	Seaweed (brown algae)	Established
Polysaccharide	Agar	Seaweed (red algae)	Established
Polysaccharide	Carrageenan	Seaweed (red algae)	Established
Polysaccharide	Chitin	Crustacean exoskeleton (waste product), algae	Established
Polysaccharide	Chitosan	Crustacean exoskeleton (waste product), algae	Established
Polysaccharide	Cellulose	Microalgae	Development stage
Polysaccharide	Starch	Microalgae	Development stage
Protein	Collagen	Fish waste (skin/bone)	Established
Protein	Gelatine	Fish waste (skin/bone)	Established
Protein	Glycoprotein (antifreeze protein)	Fish (blood)	Established
Lipid	Fish oil	Fish waste	Established
Minerals	Calcium chloride	Crustacean exoskeleton (waste product)	Industrial side product

**Table 3:** Overview of applied coating performance measurement methods.

Studied Material	Property Category	Property Details	Methodology
coating formulation	viscoelastic properties	handling application industrial feasibility	pH elastic modulus viscosity modulus solubility phase angle electrical charge
casted films	barrier properties	molecule transfer	water permeability gas permeability
coated product	barrier properties	molecule transfer	water resistance gas exchange range
coated product	coating formation properties	adhesiveness thickness penetration/absorption integrity durability	contact angle imaging analysis scanning electron microscope wettability cohesiveness
coated product	sensory properties	appearance texture odour taste	colour/opacity hardness, elasticity chewiness/gumminess
coated product	physical-chemical properties	fat oxidation water migration	water loss, water activity nitrogen degradation peroxide value malondialdehyde value total volatile basic nitrogen trimethylamine-nitrogen pH adenosine triphosphate breakdown
coated product	anti-microbial properties	safety and quality degradation	bacterial growth yeast growth mould growth

## Data Availability

No new data were created or analysed in this study.
